# Characteristics of a Dengue Outbreak in a Remote Pacific Island Chain – Republic of the Marshall Islands, 2011–2012

**DOI:** 10.1371/journal.pone.0108445

**Published:** 2014-09-30

**Authors:** Tyler M. Sharp, Andrew J. Mackay, Gilberto A. Santiago, Elizabeth Hunsperger, Eric J. Nilles, Janice Perez-Padilla, Kinisalote S. Tikomaidraubuta, Candimar Colon, Manuel Amador, Tai-Ho Chen, Paul Lalita, Jorge L. Muñoz-Jordán, Roberto Barrera, Justina Langidrik, Kay M. Tomashek

**Affiliations:** 1 Epidemic Intelligence Service, Centers for Disease Control and Prevention, Atlanta, Georgia, United States of America; 2 Division of Vector-Borne Diseases, Centers for Disease Control and Prevention, San Juan, Puerto Rico; 3 World Health Organization, Division of Pacific Technical Support, Suva, Fiji; 4 Majuro Hospital, Majuro, Republic of the Marshall Islands; 5 Division of Global Migration and Quarantine, Centers for Disease Control and Prevention, Honolulu, Hawaii, United States of America; 6 Ministry of Health, Majuro, Republic of the Marshall Islands; Centro de Pesquisas René Rachou, Brazil

## Abstract

Dengue is a potentially fatal acute febrile illness caused by four mosquito-transmitted dengue viruses (DENV-1–4). Although dengue outbreaks regularly occur in many regions of the Pacific, little is known about dengue in the Republic of the Marshall Islands (RMI). To better understand dengue in RMI, we investigated an explosive outbreak that began in October 2011. Suspected cases were reported to the Ministry of Health, serum specimens were tested with a dengue rapid diagnostic test (RDT), and confirmatory testing was performed using RT-PCR and IgM ELISA. Laboratory-positive cases were defined by detection of DENV nonstructural protein 1 by RDT, DENV nucleic acid by RT-PCR, or anti-DENV IgM antibody by RDT or ELISA. Secondary infection was defined by detection of anti-DENV IgG antibody by ELISA in a laboratory-positive acute specimen. During the four months of the outbreak, 1,603 suspected dengue cases (3% of the RMI population) were reported. Of 867 (54%) laboratory-positive cases, 209 (24%) had dengue with warning signs, six (0.7%) had severe dengue, and none died. Dengue incidence was highest in residents of Majuro and individuals aged 10–29 years, and ∼95% of dengue cases were experiencing secondary infection. Only DENV-4 was detected by RT-PCR, which phylogenetic analysis demonstrated was most closely related to a virus previously identified in Southeast Asia. Cases of vertical DENV transmission, and DENV/*Salmonella* Typhi and DENV/*Mycobacterium leprae* co-infection were identified. Entomological surveys implicated water storage containers and discarded tires as the most important development sites for *Aedes aegypti* and *Ae. albopictus*, respectively. Although this is the first documented dengue outbreak in RMI, the age groups of cases and high prevalence of secondary infection demonstrate prior DENV circulation. Dengue surveillance should continue to be strengthened in RMI and throughout the Pacific to identify and rapidly respond to future outbreaks.

## Introduction

Dengue is the most common mosquito-borne viral illness in the world, and is endemic throughout the tropics and subtropics where ∼96 million cases occurred in 2010 [1], [2]. Infection with any of four dengue viruses (DENV-1–4), which are transmitted by select *Aedes* species mosquitoes, can result in dengue, an acute febrile illness characterized by headache, body pain, retro-orbital pain, rash and leukopenia [2]. Although most DENV infections are asymptomatic or subclinical [3], ∼5% of dengue patients develop severe dengue (including dengue hemorrhagic fever [DHF] and dengue shock syndrome [4]).

Recent dengue outbreaks have been reported in the Pacific islands, including Fiji [5], Palau [6], Kiribati [7], the Federated States of Micronesia (FSM) [8]–[10], the Solomon Islands [11], and Hawaii [12], [13], with rates of attack and infection up to 6% [10] and 27% [6], respectively. Travel between the Pacific islands and dengue-endemic countries throughout the region facilitates DENV circulation, which may result in outbreaks [7]. In example of this, after an apparent absence of circulation in the Pacific Islands for many years, DENV-4 was detected in the region in 2008 and caused several outbreaks soon after [7], [14].

Dengue was apparently first detected in the Republic of the Marshall Islands (RMI) during an outbreak in 1989 in which DENV-1 was isolated from cases on Majuro, Kwajalein and Ebon atolls (U.S. Centers for Disease Control and Prevention [CDC], unpublished data). In 1990 and 2004, DENV-2 and -1, respectively, were detected in serum specimens collected from RMI residents reported to CDC as having dengue-like illness (CDC, unpublished data). In 2010, *Ae. aegypti* and *Ae. albopictus* were detected in RMI during mosquito surveys (Harry M. Savage, personal communication). Although dengue activity was not above baseline in the Western Pacific Region of the World Health Organization (WHO) in 2011, country-specific rates were highest in RMI [15].

To enable early detection of dengue and other outbreak-prone diseases, in 2009 a surveillance system was initiated in RMI that included implementation of dengue rapid diagnostic tests (RDTs) [16]. In October 2011, several RDT-positive cases were reported to the RMI Ministry of Health (MOH) from Majuro atoll. Following a rapid increase in cases, the RMI government declared a state of emergency due to the outbreak. CDC, WHO, and other partners assisted in responding to the outbreak [17]. Response activities included use of RDTs to identify dengue patients and monitor epidemiologic trends; clinical training on dengue case management according to established guidelines [2]; vector surveillance to direct public clean-up campaigns and vector control activities; and public health education regarding dengue prevention, control, and the need to seek care for dengue-like illness.

## Materials and Methods

### Site of investigation

RMI is composed of 29 atolls and five islands with a total land mass of 70 square miles (sq mi) spread across ∼750,000 sq mi of ocean (Figure S1). The 2011 population of RMI was 53,158 (759 individuals/sq mi), 70% of which resided on Majuro atoll or Ebeye island (7,413 and 80,117 individuals/sq mi, respectively) [18]. Forty percent of the population was aged ≤14 years, and the sex ratio was 102 males to 100 females.

### Investigation design

Surveillance data were collected during the outbreak, summarized weekly, and reported to WHO. After the outbreak had ended, a retrospective analysis of surveillance data was performed to: 1) describe the epidemiology of the 2011–2012 outbreak, including disease severity; 2) estimate the proportion of secondary DENV infections; 3) describe the molecular epidemiology of the DENV(s) responsible for the outbreak; and 4) identify the water containers producing vector mosquitoes.

### Data sources

Suspected cases identified at Majuro and Ebeye Hospitals were reported directly to MOH via the Dengue Surveillance Form (DSF; Figure S2A), which was implemented for the outbreak. DSF data were reported to MOH via short wave radio from all other health facilities. Case-patients’ ultimate severity of illness were captured with a second DSF (Figure S2B) that was completed upon patient discharge or follow-up evaluation.

### Diagnostic testing

Serum specimens were collected from all suspected dengue cases upon presentation to Majuro and Ebeye Hospitals and tested by RDT (Dengue Duo, Standard Diagnostics, Haryana, India). Convalescent specimens were collected upon discharge or during follow-up evaluation. For suspected dengue cases that presented to other health facilities in RMI, whole blood was collected at presentation and tested by RDT at the health facility. A convenience sample of both RDT-positive and-negative serum specimens were sent to CDC for confirmatory testing by multiplex, DENV-type-specific, real-time reverse transcriptase-polymerase chain reaction (RT-PCR) [19]. All specimens that tested negative by RT-PCR were further tested by anti-DENV IgM antibody capture ELISA (InBios International, Inc.; Seattle, WA). Unless a convalescent specimen was available, specimen diagnosis was based on the single acute specimen.

### Definitions

A suspected dengue case was an individual for whom a health care provider suspected dengue as the cause of illness and reported the case to MOH. A laboratory-positive case was a suspected dengue case for which: 1) DENV nucleic acid was detected by RT-PCR; 2) DENV non-structural protein 1 (NS1) was detected by RDT; or 3) anti-DENV IgM antibody was detected by RDT or ELISA. A laboratory-negative case was a suspected dengue case that had no laboratory diagnostic evidence of DENV infection. Dengue, dengue with warning signs, and severe dengue were defined according to 2009 WHO guidelines [2].

### Primary and secondary DENV infections

A representative sample of all laboratory-positive cases was selected to estimate the rates of primary and secondary infection. Cases were stratified by age group with optimal allocation for comparison between age groups. Sample size was calculated to estimate the proportion of secondary infections by age group based on data from the 2010 dengue epidemic in Puerto Rico [20], an error of 20%, 95% significance, and an expected 20% of specimens having insufficient specimen for testing. Of 218 selected acute (i.e., collected within five days of illness onset) specimens, 194 (89%) were tested at a dilution of 1∶100 to detect anti-DENV IgG antibody by ELISA using DENV-1–4 antigen and a cut-off value of OD_450_≥0.15 [21], [22]. Secondary infection was defined by detection of anti-DENV IgG antibody in a laboratory-positive acute specimen. Primary infection was defined by lack of detection of anti-DENV IgG antibody in a laboratory-positive acute specimen.

### Sequencing and phylogenetic analysis

Serum specimens in which DENV nucleic acid was detected and with CT values <25 were inoculated into cultures of C6/36 cells, in which growth of DENV was detected by RT-PCR and indirect immunofluorescence [23]. Viral RNA was extracted from culture supernatant using the Universal BioRobot System (Qiagen; Valencia, CA). The envelope glycoprotein (E) gene (1,485 base pairs) was successfully amplified and sequenced in one specimen. Multiple sequence alignment with 39 other Indonesian genotype strains obtained from GenBank was performed using ClustalW available in MEGA 5 (megasoftware.net). GTR+Γ+I4 was selected as the best nucleotide substitution model by MODELTEST v3.7. Phylogenetic relationship was inferred by 60 million Markov Chain Monte Carlo run in BEAST v1.7.5 (beast.bio.ed.ac.uk) under Bayesian skyline prior assuming a relaxed lognormal molecular clock. All ESS values reported >400. A Bayesian maximum credibility tree was constructed in TreeAnnotator (found in the same BEAST package) and visualized in FigTree v1.3.1. Posterior probabilities were used as statistic for internal nodes. Maximum likelihood phylogenetic trees with 1,000 bootstrap replicates were also inferred using MEGA 5; both trees rendered nearly identical tree topologies, confirming genetic relatedness. Genotype was referred to by previously described nomenclature [24], [25].

### Entomology

Relative abundance and species composition of juvenile mosquito populations in aquatic container habitats were assessed in 155 households on Majuro atoll during 3–8 November, 2011. Sampling focused on four communities (Rita, Uliga, Long Island and Laura) where the incidence of suspected dengue was high during 10 October–5 November 2011. All outdoor container habitats at selected households were inspected for the presence of standing water, juvenile mosquitoes, and if >20 pupae were present in the container. Mosquito specimens were retained from a subset of samples from each container habitat class and preserved in ethanol (larvae) or reared to eclosion (pupae) for identification to species.

### Statistical analyses

All surveillance data were entered into a Microsoft Access (Microsoft Corporation, Redmond, WA) database at MOH. Rates of suspected and laboratory-positive dengue cases were calculated using population denominators from the 2011 RMI Census [18]. Unless otherwise stated, statistical differences in proportions were evaluated using the Chi-squared test. The nonparametric equality-of-medians test was used to compare median age between groups. Relative risk ratios (RR) were used to calculate differences between effect sizes. Graphs were produced in Microsoft Excel (Microsoft Corp., Redmond, WA). Statistical calculations were performed in EpiInfo7 (CDC, Atlanta, GA).

### Ethics statement

This investigation underwent institutional review at CDC and was determined to be public health practice and not research; as such, Institutional Review Board approval was not required. All specimens tested in RMI were not anonymized to facilitate reporting of diagnostic test results to clinicians. All specimens sent to CDC were anonymized prior to shipment.

## Results

### Case identification

**Figure 1:**
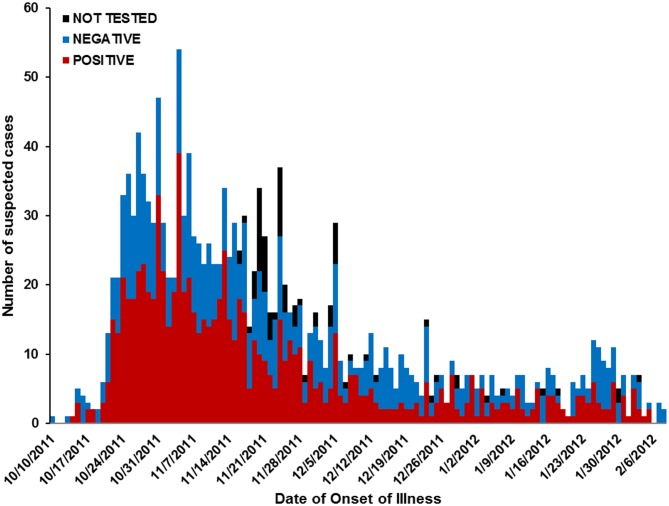
Epidemic curve of suspected dengue cases by date of illness onset in the Republic of the Marshall Islands, October 2011–February 2012. Laboratory case definition and date of illness onset were compiled as indicated. Only one date per week is shown on the x-axis.

The first identified laboratory-positive case had onset of illness on October 14, 2011 and resided in Majuro atoll (Fig. 1). The number of suspected cases began to increase steeply one week later and peaked on November 4. Cases continued to be identified at comparatively low levels through the first months of 2012, and the outbreak was declared over in February. A median of 8 (range: 0–54) suspected cases were identified per day during the outbreak.

### Diagnostic testing

Serum specimens were collected upon presentation from all patients with dengue-like illness. Specimens were collected a median of two days (25^th^–75^th^ quartile: 1–3) post-onset of illness. Paired acute and convalescent specimens were available for 59 (3.7%) cases.

Of 1,603 suspected dengue cases identified during the outbreak (3.0% of RMI residents), 657 (41%) were laboratory-negative, 79 (4.9%) were not tested, and 867 (54%; 1.6% of RMI residents) were laboratory-positive. Of 1,379 specimens tested with the RDT in RMI, 626 (45%) were positive: 536 (86%) by detection of NS1, 73 (12%) by detection of anti-DENV IgM antibody, and 17 (2.7%) by detection of both NS1 and anti-DENV IgM antibody. From a convenience sample of 845 RDT-positive and -negative specimens sent to CDC and tested by RT-PCR, DENV-4 was detected in 482 (57%) and was the only DENV detected during the outbreak. Of the 363 specimens that tested negative by RT-PCR and were further tested by IgM ELISA at CDC, anti-DENV IgM antibody was detected in 45 (12%).

### Epidemiologic findings

**Figure 2:**
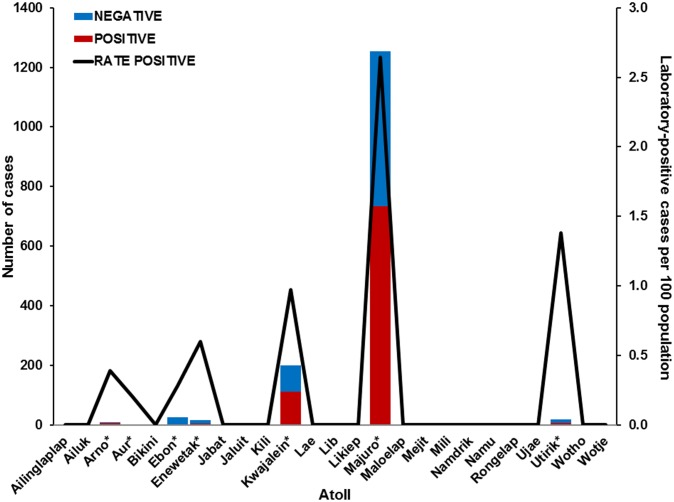
Atoll of residence of suspected dengue cases identified in the Republic of the Marshall Islands, October 2011–February 2012. Laboratory case definition and atoll of residence were compiled as indicated. Asterisks indicate atolls with laboratory-positive cases. Ebeye island is located on Kwajalein atoll.

Laboratory-positive cases were identified from seven of the 25 inhabited atolls and islands that comprise RMI (Fig. 2). Among these regions, the median rate of suspected and laboratory-positive cases per population was 2.4% (range: 0.1–4.6%) and 0.6% (range: 0.2–2.7%), respectively; both were highest in Majuro atoll.

**Figure 3:**
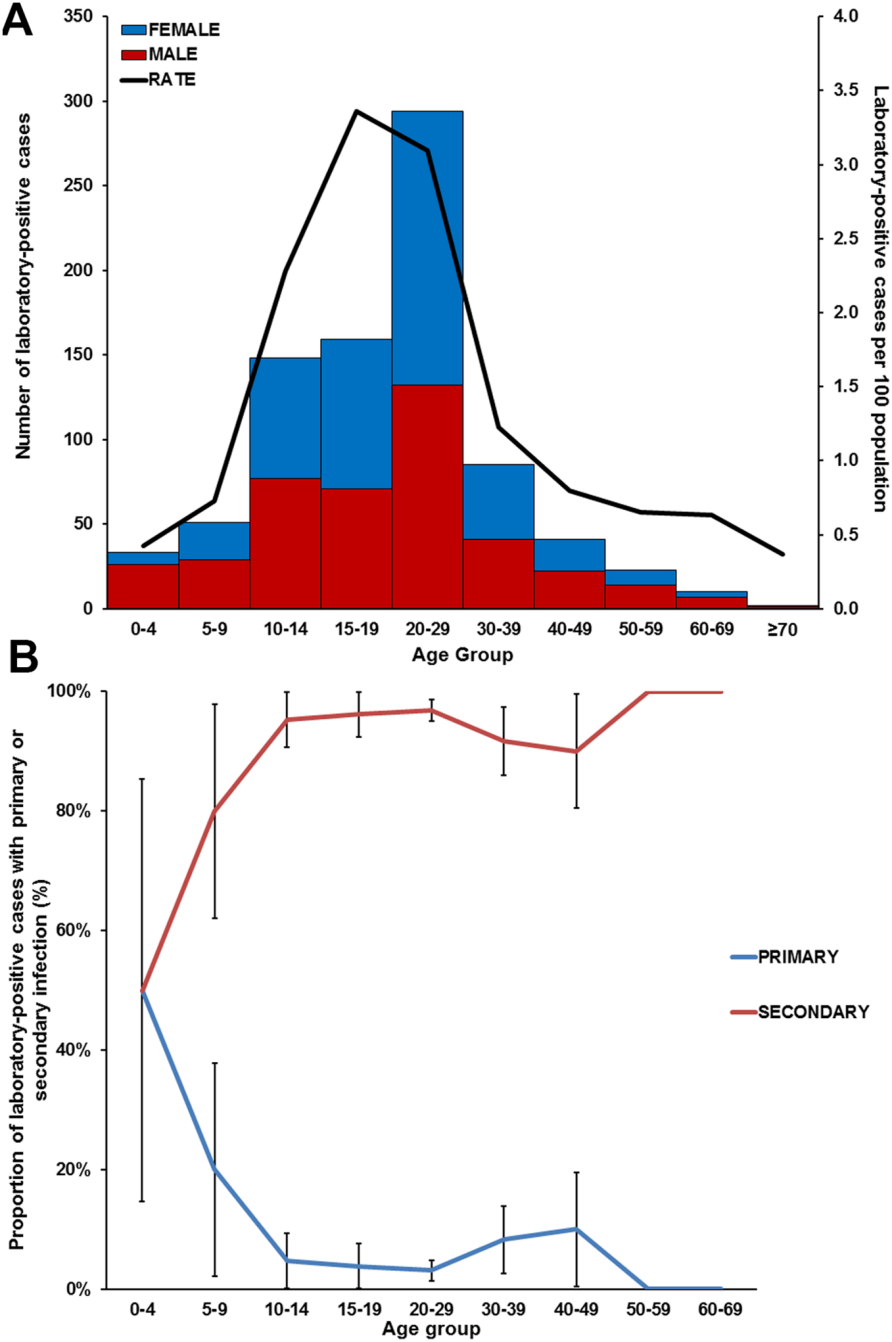
Age distribution of laboratory-positive dengue cases in the Republic of the Marshall Islands, October 2011–February 2012. **A**: Age distribution by sex and rate of laboratory-positive cases; **B**: Primary and secondary DENV infections by age group from a representative sample of all laboratory-positive cases; error bars indicate standard error.

Most (72%) laboratory-positive cases were aged 10–29 years (Fig. 3A). The highest rate of laboratory-positive cases per population (3.4%) was in individuals aged 15–19 years, and lowest (0.4%) in individuals aged 0–4 or ≥70 years. Males and females were equally represented in all age groups except 0–4 year-olds (p<0.001), in which 79% of laboratory-positive cases were male.

### Primary and secondary DENV infections

From a representative sample of 194 laboratory-positive acute specimens, 184 (95%) had evidence of secondary infection (Fig. 3B). Rate of secondary infection by age group was 50% in 0–4 year-olds, and increased with age group until a plateau of ∼90% was reached in 10–14 year-olds.

### Molecular epidemiology

**Figure 4:**
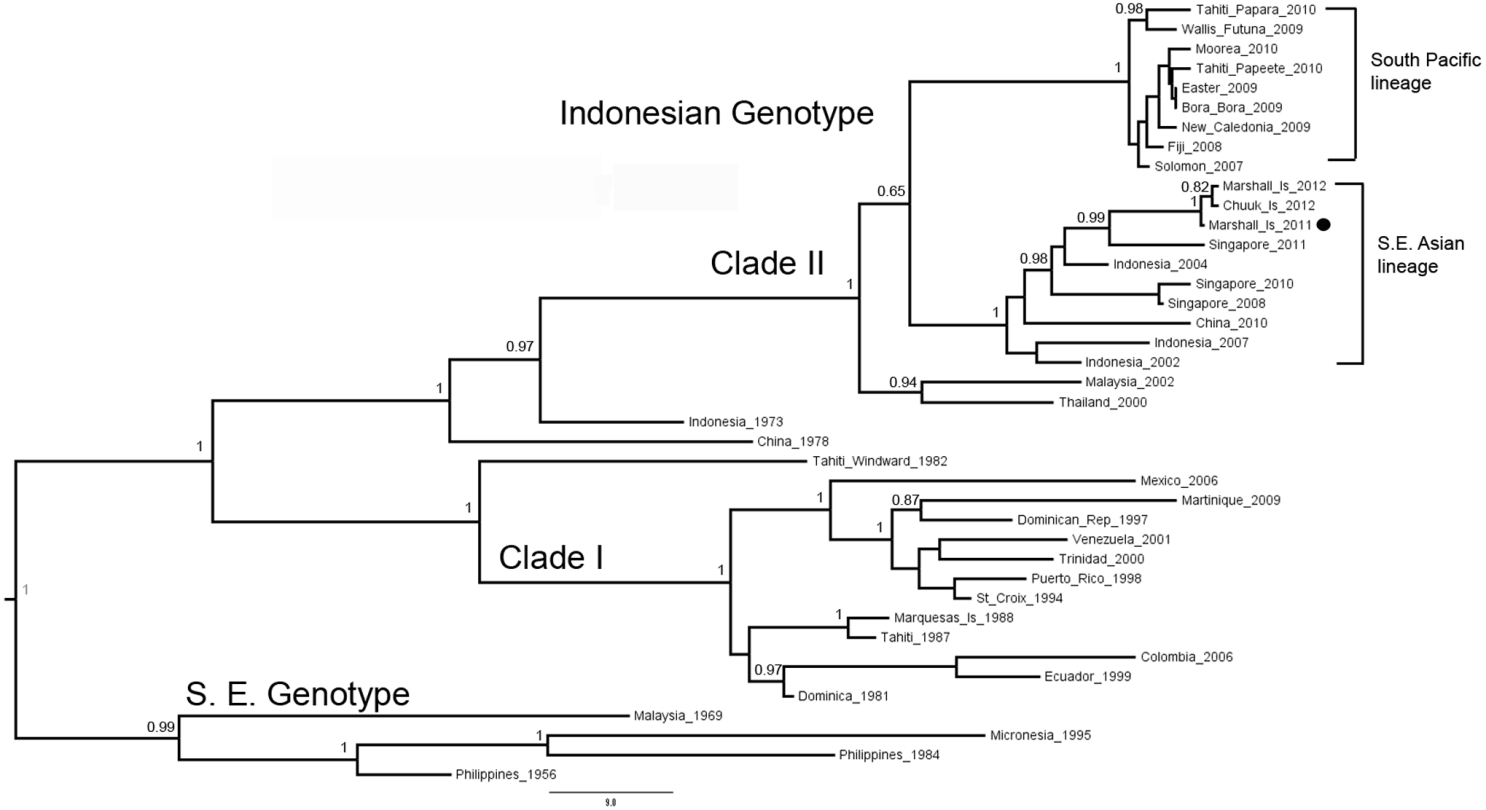
Bayesian maximum clade credibility phylogenetic tree of DENV-4 detected during the 2011–2012 dengue outbreak in the Republic of the Marshall Islands. N = 40 E gene sequences (1,485 basepairs). Internal nodes supported by posterior probability. The dot indicates the DENV-4 isolated from the Republic of the Marshall Islands in 2011 (GenBank accession number JX891655).

Following virus isolation and E gene sequencing, Bayesian phylogenetic analysis demonstrated that the virus circulating in RMI is distinct from other DENV-4 previously identified in the Pacific islands, and instead belongs to clade II of the Indonesian genotype that is geographically associated with Southeast Asia (Fig. 4). The most closely related DENV-4 was isolated from cases that occurred in 2012: an individual from Japan with travel history to RMI, and a resident of Chuuk, FSM. Other closely related viruses were detected in Singapore in 2011 and Indonesia in 2004.

### Disease severity

Laboratory-positive cases were not significantly associated with sex, age, or ethnicity (Table 1). Although more laboratory-positive (79%) than laboratory-negative (75%) cases met the 2009 WHO dengue case definition [2], this difference only approached statistical significance (p = 0.06). Laboratory-positive case-patients were 1.8 (95% confidence interval [95% CI]: 1.3–2.5) times more likely to be hospitalized than laboratory-negative case-patients (p<0.001). Laboratory-positive case-patients had dengue with warning signs significantly more frequently than laboratory-negative case-patients (p = 0.02). The most frequently identified warning signs among laboratory-positive case-patients were persistent vomiting (35%) and abdominal pain (59%); the latter was significantly more common in laboratory-negative cases (75%; p = 0.007). Fluid accumulation (e.g., pleural effusions, ascites) was only observed in laboratory-positive cases (Fischer’s exact test, p = 0.16). Concomitant rise in hematocrit and decrease in platelets was 4.3 times (95% CI: 2.2–8.4) more likely to occur in laboratory-positive than laboratory-negative case-patients (p<0.001). Six (0.7%) laboratory-positive case-patients had severe dengue. No fatal cases were reported.

**Table 1:** Demographic and clinical characteristics of suspected dengue cases reported during an outbreak in the Republic of the Marshall Islands, October 2011–February 2012.

Characteristic	All cases*	Laboratory-positivecases	Laboratory-negativecases
	N = 1,603	N = 867	N = 657
**Sex**, % male†	750 (48)	420 (50)	289 (45)
**Age**, medianyears (range)	20(0 days–89 years)	20(0 days–86 years)	20(0 days–89 years)
**Ethnicity**,‡ n (%)			
Pacific IslanderAsianCaucasianOther	1,294 (97)37 (2.8)2 (0.1)6 (0.4)	677 (97)21 (3.0)1 (0.1)2 (0.3)	553 (97)15 (2.6)1 (0.2)4 (0.7)
**Dengue**, n (%)	1,241 (77)	688 (79)	495 (75)
**Hospitalized**, n (%)	160 (10)	**110 (13)**	**47 (7.2)**
**Hemorrhage**, n (%)	84 (5.2)	40 (4.6)	42 (6.4)
**Dengue with warning signs**, n (%)Abdominal painPersistent vomitingFluid accumulationMucosal bleedLethargyLiver enlargementRise in hematocrit with decrease in platelets	312 (19)205 (66)99 (32)5 (1.6)22 (7.1)50 (16)6 (1.9)69 (22)	**187 (22)** **111 (59)**66 (35)5 (2.7)14 (7.5)34 (18)4 (2.1)**57 (30)**	**111 (17)** **83 (75)**32 (29)0 (0)7 (6.3)13 (12)2 (1.8)**10 (9.0)**
**Severe dengue**, n (%)Severe plasma leakageSevere bleedingSevere organ impairment	9 (0.6)2 (22)5 (56)2 (22)	6 (0.7)1 (17)4 (67)1 (17)	3 (0.5)1 (33)1 (33)1 (33)

Numbers in bold indicate a significant difference in proportions (Chi-squared, p<0.05) between laboratory-positive and -negative cases.

*79 cases were not tested.

† Sex was not reported for 34 cases.

‡ Ethnicity was not reported for 264 cases.

### Maternal and infant cases

Four infants aged <1 year tested positive with the RDT: three by detection of NS1, and one by detection of anti-DENV IgM antibody; all were hospitalized. One infant was a neonate male born to a febrile mother in whom NS1 was detected on the day of parturition. On day seven of life, the infant developed severe dengue with cyanosis and severe bleeding requiring blood transfusion. Both the baby and mother recovered and were ultimately discharged. As 111 pregnant women gave birth during the outbreak, the rate of documented vertical DENV transmission resulting in dengue during the outbreak was 0.9% (1/111). Four additional pregnant, laboratory-positive females were identified during the outbreak; all recovered without complication. No reported fetal losses or perinatal deaths were associated with maternal DENV infection during the outbreak.

### Atypical clinical cases

An 11-year-old boy from Utrik atoll had onset of fever in early November and subsequently had streaks of blood in his stool. After being transported to Majuro Hospital, he was given intravenous ampicillin, cephazoline, and metronidazole for a presumptive diagnosis of typhoid fever; however, his fever persisted. White blood cell count, platelet count, hemoglobin, and hematocrit were normal. NS1 was detected on day 8 of illness, and *Salmonella* typhi was identified in a blood culture the following day. He was discharged home in stable condition after seven days of hospitalization.

An 18-year-old male in his third month of treatment for leprosy was admitted with a two-day history of fever and malaise. On examination he was febrile with patchy nodular skin lesions on the trunk and extremities. NS1 and DENV-4 were detected in a serum specimen. His hospital course was unremarkable and he continued treatment for leprosy, including a decreased dose of prednisone. He was discharged on day six of hospitalization.

### Entomologic findings

Mosquito larvae or pupae were detected at 87% of inspected homes (i.e., House Index) and in 64% of containers with standing water outside homes (i.e., Container Index), with an estimated density of 413 mosquito-positive containers per 100 households (i.e., Breteau Index) (Table 2). Indoor inspections were performed at a subset of selected households; however, water-filled containers were rarely observed and all were negative for juvenile *Aedes* spp. mosquitoes (data not shown). Large water storage receptacles were the most abundant water-filled container (235 per 100 households inspected), and accounted for 32% of mosquito-positive containers and 50% of containers with >20 pupae. Discarded tires, domestic utensils (e.g., washing or cooking items, trash containers) and small refuse items (e.g., cans, disposable food packaging) collectively represented 44% of mosquito-positive containers and 29% of containers with >20 pupae.

**Table 2:** Abundance and distribution of juvenile mosquitoes in water-filled containers identified during household surveys (N = 155) on Majuro atoll, Republic of the Marshall Islands, 3–8 November, 2011.

Container Class(representative types)	Number of Containers per 100 Households (% of Water-filled Containers Inspected)		
	With Water	Larvae or Pupae Present	>20 Pupae Present
**Water Storage**(tanks, cisterns, drums)	235	133 (57)	17 (7)
**Discarded Tires**	76	67 (88)	5 (7)
**Domestic Utensils in Use**(buckets, cooking pots,wash tubs, garbage cans)	105	58 (56)	4 (14)
**Containers with Plants**(planter pots, vases)	28	21 (75)	2 (7)
**Small Refuse**(bottles, cans, food packaging)	83	55 (67)	1 (1)
**Natural Containers**(coconut shells, tree holes, leaf axils)	46	38 (82)	1 (3)
**Large Refuse**(discarded appliances,furniture and vehicles)	23	16 (69)	1 (3)
**Miscellaneous Items**(animal watering pans,tarps, boats, wells)	54	24 (45)	3 (5)
Total	650	413 (64)	34 (5)

**Figure 5:**
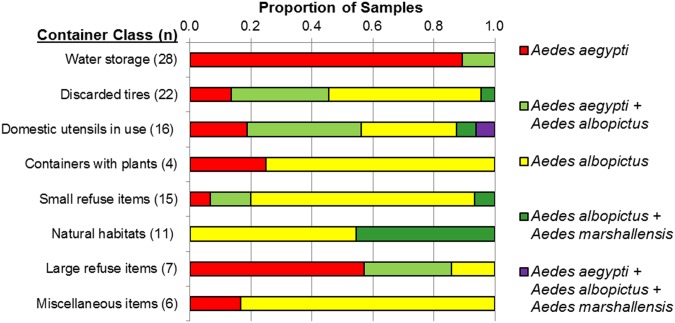
*Aedes* species composition in juvenile mosquito samples collected from water-filled containers on Majuro atoll, Republic of the Marshall Islands, 3–8 November, 2011. Examples of habitat types in each container class are provided in Table 2.

A total of 738 *Aedes* spp. juveniles collected from 109 containers with larvae or pupae were identified to species (Fig. 5). Specimens of *Ae. aegypti* were identified in all juvenile mosquito samples collected from water storage containers, either alone or co-occurring with *Ae. albopictus*. *Ae. albopictus* was the most frequently identified species in samples collected from all other container classes except large refuse items. Specimens of *Ae. marshallensis* were rarely collected from artificial container habitats, but were present in almost half of samples from natural container habitats concurrently with *Ae. albopictus*. *Culex* spp. larvae and pupae specimens were present in 13 samples, with the greatest frequency in discarded tires (6 [27%] of 22 samples).

## Discussion

The epidemiologic characteristics of this outbreak were captured by improving public and clinical awareness of dengue, enhancing surveillance to enable rapid case reporting, and use of a RDT followed by confirmatory testing to define dengue cases. Pertinent findings include 3% of all RMI residents being reported as a suspected dengue case, laboratory confirmation of dengue in 3% of Majuro residents, hospitalization of 13% of dengue cases, and identification of water storage containers as prominent sources of vector mosquitoes. The prevalence of secondary infection in all age groups demonstrates that DENVs regularly circulate in RMI.

The rate of suspected dengue cases observed during this outbreak (3%) was similar to that recently reported from FSM and the Solomon Islands [8], [11], but was not as high as the 10–50% reported during “virgin soil” outbreaks in Tonga in 1975 [26] and Niue in 1985 [27]. Moreover, the rate of severe dengue (0.6%) was not particularly high, and no fatal cases were detected. Rates of DHF and fatal dengue in prior outbreaks in the Pacific ranged from 2–8% [5], [9] and 0.4–1.2% [5], [26], respectively. Possible explanations for apparent differences in both the attack rate and rate of severe case detection include dissimilarities in: prior DENV transmission, which presumably had not occurred in Tonga and Niue but did in RMI, and consequent differences in immunologic susceptibility of the population; sequence of and time between patients’ DENV infections, which can affect disease severity [2], [28]–[30]; and most dengue patients having access to medical care at Majuro Hospital and seeking care early, which likely improved identification of dengue cases from across the clinical spectrum and may have reduced the number of severe dengue cases, respectively. Last, establishing dengue wards in Majuro Hospital and training clinicians in the current best practices in dengue patient management may have prevented fatal cases, as has been previously reported [31].

Several studies have demonstrated that primary infection with DENV-4 infrequently causes clinically apparent illness [20], [32]–[37]. Consistent with these observations, the overwhelming majority of cases in this investigation were experiencing secondary infection with DENV-4. During a community survey conducted during a DENV-4 outbreak in FSM in 1995, 6% of residents experienced a dengue-like illness, anti-DENV IgM seroprevalence was 6–36%, and nearly all residents had been previously infected with a DENV [10]. Although anti-DENV seroprevalence was not determined during the RMI outbreak, the findings from FSM and elsewhere suggest that many primary infections may have occurred in individuals in RMI who did not develop clinically apparent illness, suggesting a higher rate of DENV infection than was documented. Moreover, the high rate of clinically apparent dengue cases in individuals aged 10–29 years, nearly all of which were secondary infections, compared to the rate in individuals aged <10 years suggest a dengue outbreak occurred in RMI 10–15 years ago. This is in agreement with anecdotal reports from clinicians at Majuro Hospital of an outbreak of dengue-like illness in the early 2000s (MOH, unpublished data). Finally, the observation that >70% of dengue case-patients aged 0–9 years were experiencing secondary infection demonstrates that DENVs regularly circulate in RMI. Therefore, dengue cases occurring before the 2011–2012 outbreak may have been overlooked due to a lack of surveillance for and/or clinical awareness of dengue.

Interestingly, the DENV-4 that caused the RMI outbreak was distinct from other DENV-4 circulating previously in the Pacific, but was nearly identical to viruses isolated from dengue patients in 2012 from FSM and another with travel history to RMI. Moreover, the genetic relatedness of the DENV-4 from RMI to DENV-4 from Southeast Asia suggests that human travel from Southeast Asia resulted in the RMI outbreak, which subsequently spread to FSM. A similar chain of events enabled spread of DENV-4 throughout Pacific islands in 2009 [7].

Although *Ae. aegypti* was likely the primary mosquito vector responsible for DENV transmission during the RMI outbreak, *Ae. albopictus* is also a competent vector and may have contributed to DENV transmission, as was recently suggested to be the case in FSM [38]. First detected in RMI from a single container habitat on Majuro Atoll in 1981 [39], *Ae. albopictus* has since been collected from multiple locations within RMI (Harry M. Savage, personal communication). Our results confirm that this species is now well established on Majuro atoll, representing the vector species most frequently recovered from domestic container habitats other than large water storage and refuse items. *Ae. marshallensis* may also have contributed to DENV transmission, although the vector competence of this species is unclear [40], [41].

The high abundance of water storage containers infested with *Ae. aegypti* suggests they were the most important source of vector mosquitoes. Since only 5% of households in RMI have access to piped water, 79% of households store rainwater and the remainder rely on well or bottled water [18]. Conventional (e.g., cisterns) and ad-hoc (e.g., re-purposed chest freezers) water storage containers frequently lacked screens or tight-fitting covers to preclude mosquito access. In contrast, 1,500 gallon water catchment systems had been recently installed in some households [18] and had tight-fitting lids or mesh screens sealing all entry points; no mosquito juveniles were detected in such containers. These findings emphasize the importance of designing and managing water storage containers that are inaccessible to mosquitoes, and highlight the benefits of cooperative programs for improving water collection and storage. Such improvements should be paired with householder education to ensure sustainability [42]. The high colonization rate of discarded tires by vector mosquitoes, particularly *Ae. albopictus*, was similar to observations from a recent dengue outbreak in FSM [8]. Sustainable dengue prevention strategies should incorporate long-term solutions to manage solid waste and outreach to householders on the importance of preventing mosquito development around their home.

This investigation was subject to several limitations. Because confirmatory diagnostic testing was not performed for nearly half of suspected cases that were tested only by RDT, which may yield false-negative test results [43], the true incidence of dengue was likely higher than was reported here. Nonetheless, because nearly half of suspected dengue cases had no evidence of DENV infection and abdominal pain was more common in laboratory-negative cases, the etiology of non-dengue causes of acute febrile illness (e.g., typhoid fever [44], leptospirosis [45], rickettsioses [46], other arboviruses [47]) should be further investigated in RMI to enable appropriate patient management. Also, vector surveillance data were not collected after application of pesticides, and thus we were unable to evaluate the efficacy of vector control activities in controlling either juvenile or adult mosquito populations. Finally, because it is unclear if other flaviviruses have circulated previously in RMI, some individuals defined as experiencing secondary infection may have been misclassified due to antibody cross-reactivity between flaviviruses [48].

This outbreak demonstrated the acute burden that a dengue outbreak can have in a Pacific island population. To reduce the likelihood of future outbreaks, evidence-based behavioral and interventional approaches to reduce mosquito vector populations should be developed and routinely implemented. Dengue surveillance should continue to be strengthened in RMI and other Pacific island countries to rapidly detect and respond to outbreaks. In anticipation of a dengue vaccine, the age group of individuals affected by dengue in the Pacific should be elucidated to better inform vaccine campaigns.

## Supporting Information

Figure S1 **Map depicting the geographic location of the Republic of the Marshall Islands.** Source: http://www.travelpod.com/bin/graphics/maps/country/large/mh-map.gif. Published under the Creative Commons-BY license.
(TIF)

Figure S2 **Dengue Surveillance Forms used to capture demographic, clinical and laboratory data for suspected dengue cases reported to the Ministry of Health during a dengue outbreak in the Republic of the Marshall Islands, October 2011–February 2012. A**: Surveillance form used to capture data at presentation. **B**: Surveillance form used to capture data at discharge or during follow-up evaluation.
(TIF)
