# Supplementary figures and images for: Characteristics of a Dengue Outbreak in a Remote Pacific Island Chain – Republic of the Marshall Islands, 2011–2012
Source: PLoS One. 2014 Sep 30;9(9):e108445. doi: 10.1371/journal.pone.0108445 (PMC4182480; doi:10.1371/journal.pone.0108445)

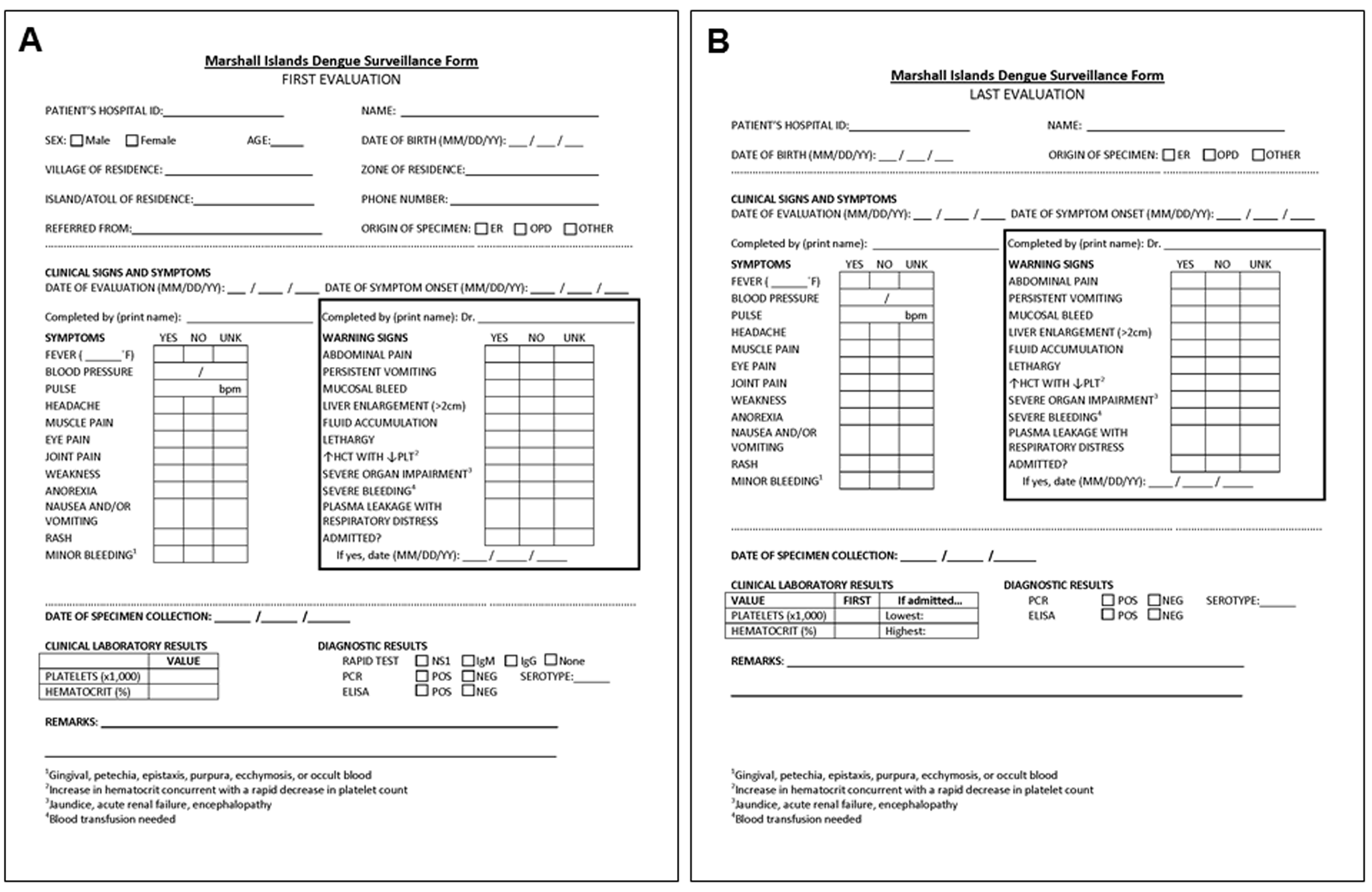

Supplement: Figure S2 — Dengue Surveillance Forms used to capture demographic, clinical and laboratory data for suspected dengue cases reported to the Ministry of Health during a dengue outbreak in the Republic of the Marshall Islands, October 2011–February 2012. A: Surveillance form used to capture data at presentation. B: Surveillance form used to capture data at discharge or during follow-up evaluation. (TIF) [file pone.0108445.s002.tif]
